# Sabina Faiz Rashid: research on community resilience

**DOI:** 10.2471/BLT.20.030720

**Published:** 2020-07-01

## Abstract

Sabina Faiz Rashid talks to Andréia Azevedo Soares about anthropology, poverty, inequality and sex education in Bangladesh.

Q: How did you come to focus on gender and health issues?

A: When I was 24 years old, I spent six months in a village called Uddomdi in south eastern Bangladesh where I had a number of encounters with girls and women that made me want to better understand their multifaceted lives. A few years later, in 1996, I returned to do research on a group of women who were using a contraceptive implant that was negatively affecting the health of some of them. Those experiences were transformative.

Q: In what way?

A: Well, for one thing, I was deeply affected by the kindness and hospitality of the communities, but I was also exposed to much that was entirely new to me. I grew up in many different countries because my father was a diplomat, but I had never encountered that level of poverty, nor had I ever lived in a village. The experiences not only changed my naive perceptions of the poor, they gave me insights into the impact of gender on choice and agency and the extent to which structural and social determinants of poverty influence health. Uddomdi challenged me to rethink many of my assumptions. I had naively assumed that I was going to ‘explain things’ and ‘bring solutions’ to the local people’s problems. I soon realised that their knowledge and practices were often based on pragmatic decisions taken in the face of significant deprivation. In fact, I was struck by the community’s resourcefulness and resilience. Moreover, many of my recommendations would have been difficult to implement given the resources available. So, the experiences were an invaluable lesson in humility and the importance of community-centric research, by which I mean research conducted *with* communities rather than *on* them.

Q: You studied anthropology at university in Australia. How did that background inform your public health work?

A: There are many ways in which anthropology feeds into public health and the evidence generated through an anthropological approach can be used to improve research queries and even the design of programmes, making them more culturally appropriate and gender sensitive. To give you an example, during one of the floods in Bangladesh, I conducted research on female adolescents and their menstrual practices. Part of that work examined the ways in which taboos associated with menstruation and the lack of privacy in flood shelters or relief camps made it difficult for the girls to wash their menstrual cloths or to frequently change them. This resulted in them suffering from anxiety, shame and infections. The issue of cultural constructs and the shame deriving from them – the *impact* of that shame on young women’s experience of menstruation – had not even been considered. However, such things are clearly of vital importance and need to be accounted for in the design of menstrual health interventions for adolescent girls.

“There are many ways in which anthropology feeds into public health.”

Q: Sexual and reproductive health right have been at the core of your work with BRAC university. What do you consider your main achievements?

A: The establishment of the Centre of Excellence for Gender, Sexual and Reproductive Health Rights would have to be one. The centre was set up in 2008 and has developed over the years to carry out a wide range of research, policy, advocacy and training activities. Since 2014, the centre has been running training programmes aimed at different groups of professionals, with topics including health rights, consent and choice, sexuality, sexual diversity, masculinity, and so on. Government front-line health workers are among the groups we have trained. For most participants, it is the first time they have been introduced or exposed to such topics. Workshops and training programmes are delivered using films, animations, and photography and are followed by discussions. Our aim is to promote understanding and conceptual clarity through a human rights-based framework.

Another headline achievement for the centre is getting the topic of sexuality, including sexual diversity, in the National Institute of Population Research and Training (NIPORT) educational curriculum in 2018.

Q: Can you say more about that?

A: NIPORT is an autonomous national research institute that works on family planning and trains the 85 000 family welfare assistants and volunteers who are employed by the government to disseminate sexual and reproductive health information and services door-to-door. It is important to bear in mind that section 377 of the Bangladesh Penal Code still criminalizes "unnatural" sex, so the inclusion of this content in the NIPORT curriculum was a huge step.

Q: How does the NIPORT curriculum address issues such as sexual orientation?

A: The curriculum defines the terms sexuality and sexual diversity and explains that sexual orientation or desire varies and that people with different orientations are not bad or mentally ill. The content also clearly defines the terms homosexuality, bisexuality, heterosexuality and intersexuality. The content was developed by the centre with the assistance of technical advisors from Creating Resources for Empowerment in Action, a feminist human rights organisation based in New Delhi, India.

Q: Bangladesh has committed to eliminating child, early and forced marriage by 2030, in line with sustainable development targets. Is this achievable in Bangladesh?

A: I have my doubts. According to the 2017 Child Marriage Restraint Act, the minimum legal age for marriage in Bangladesh is 18 years for girls and 21 for boys. However, the law gives courts discretion to allow child marriages in “special cases”, while neglecting to define what those “special cases” are. It should be noted that not all early marriages are necessarily coerced or forced. In some cases, young people meet and run away and get married. Girls may drop out of school, be unemployed, be fearful of crime and insecurity, poverty – all these factors increase the likelihood of early marriage. And now with COVID-19 increasing job losses and making the lives of the poorest even more insecure, I don’t expect to see a decline in early marriages.

Q: The James P Grant School of Public Health is currently conducting rapid surveys and qualitative studies to assess the impact of COVID-19 on different communities. What are the preliminary findings?

A: We are currently conducting 24 rapid research assessments with different population groups, including health workers, garment workers, informal settlement residents, rural poor, migrants, transgender communities and frontline workers, to name just a few. The initial results paint an alarming picture. Frontline health workers report that the quality of personal protective equipment supplied to them needs to be improved and complain of physical exhaustion and fear of the virus. Most of the poor are worried about food consumption and future jobs. A multi-phase survey, which focused on nutrition, income, gender and mental health shows that most households surveyed are suffering a complete loss of income with people surviving on lentils, rice and potatoes, and that 58% (759 of 1309) of individuals surveyed report higher levels of stress. The shutdown has also severely impacted the 22 transgender people we surveyed. They reported a sharp drop in income for sex work, as well as being stigmatised as carriers of the virus. Many do not benefit from the various relief initiatives of government bodies. Most of these problems were exacerbated by the unprecedented lockdown that was implemented by the government until the end of May.

“The shutdown […] resulted in most of the population involved in informal labour losing income.”

Q: How has the lockdown affected people in Bangladesh?

A: The problem is that we have adopted a model from countries that have better social safety nets and support structures. According to our research, the shutdown that was implemented in Bangladesh resulted in most of the population involved in informal labour losing income, giving rise to serious problems, including hunger. Furthermore, most of the urban poor struggled to follow the precautionary guidelines of distancing, handwashing with soap and wearing masks, given the high population density, lack of available water and toilet facilities, and costs of purchasing these items. Our research also documented other challenges, such as increased violence and stress. On top of everything else, due to the widespread stigma and fear surrounding COVID-19, some households were forcibly removed from the slums and rural villages.

Q: So how should the pandemic response be different?

A: We need to pay more attention to unintended consequences of public health interventions and recognize that population health is driven by multiple determinants rather than by a single biomedical factor – in this case the novel coronavirus. While doing everything you can to limit the virus’ spread may make perfect sense at the aggregate level, you have to take into account the structural, socio-economic and health conditions of those communities living in deplorable conditions. The social determinants of health have become part of main stream public health discourse, but we still tend to blame the poor rather than recognise the very real limitations poverty places on them and the health risks to which they are exposed.

I didn’t fully appreciate the importance of the social determinants of health until I started working in and with the communities I was supposed to be helping in the late 90s. A turning point came for me when I was doing my PhD research with married adolescent women in 2001. I was asking them about reproductive health experiences and services, but their responses inevitably came back to worries about jobs, food insecurity, slum evictions, etc. Over time, I realised that they were telling me that their health could not be separated from their other concerns, that their health was tied in with the structural, social, economic and political realities of their lives. To realise the health rights for the most vulnerable we need to acknowledge these realities and take them into account when drafting and implementing public health policy.

